# A Sweet Killer: Mesoporous Polysaccharide Confined Silver Nanoparticles for Antibacterial Applications

**DOI:** 10.3390/ijms12095782

**Published:** 2011-09-09

**Authors:** Robin J. White, Vitaly L. Budarin, James W.B. Moir, James H. Clark

**Affiliations:** 1 Green Chemistry Centre of Excellence, Department of Chemistry, University of York, Heslington, York, YO10 5DD, UK; E-Mails: vlb1@york.ac.uk (V.L.B.); jhc1@york.ac.uk (J.H.C.); 2 Department of Biology (Area 10), University of York, PO Box 373, York, YO10 5YW, UK; E-Mail: james.moir@york.ac.uk

**Keywords:** antibacterial, mesoporous, nanoparticles, nanotechnology, polysaccharide, silver, starch

## Abstract

Silver nanoparticles (AgNP) confined within porous starch have been prepared in a simple, green and efficient manner, utilising the nanoporous structure of predominantly mesoporous starch (MS) to act as nanoparticle stabiliser, support and reducing surface. MS/AgNP materials present high surface areas (*S*_BET_ > 150 m^2^ g^−1^) and mesopore volumes (*V*_meso_ > 0.45 cm^3^ g^−1^). The interaction of the AgNP precursor and forming nanoparticle nuclei with the mesoporous domains of the porous polysaccharide, direct porosity to increasingly narrower and more defined pore size distributions, indicative of a degree of cooperative assembly. Transmission electron microscopy images indicated the presence of spherical AgNP of a size reflective of the porous polysaccharide mesopore diameter (e.g., 5–25 nm), whilst XPS analysis confirmed the metallic Ag^0^ state. Materials were prepared at relatively low Ag loadings (<0.18 mmol g^−1^), demonstrating excellent antimicrobial activity in solid and liquid phase testing against Gram negative (*E. coli*) and positive (*S. aureus*) model bacteria. The resulting materials are biocompatible and present a useful solid porous carbohydrate-based polymer vehicle to control the AgNP size regime and facilitate transference to a biological environment.

## 1. Introduction

The use of green chemistry principles and renewables (e.g., polysaccharides) is ever more important in the rapidly developing field of nanotechnology [1]. The general aim being the total elimination or minimisation of waste and the implementation of the twelve green chemistry principles [2–4] in materials preparation, in terms of using synthetic route design, non-toxic chemicals, environmentally benign solvents, and renewable materials. In this context, the core of future technological progress will be based upon the increasing ability to manipulate matter at the nanometre scale, whilst attempting to emulate the activity of natural nanoscale systems [5].

Metallic nanoparticles (MNP) are chemical entities displaying higher chemical activity and interaction specificity compared to their organic counterparts. Electronic state quantisation of the MNP structure, and manipulation of these states via size and shape control, allows direction for specific applications. One route to achieve this control is to fuse nanoporous and nanoparticle technology via materials presenting pore diameters in the micro (<2 nm) and mesoporous ranges (>2, <50 nm). Nanoparticle immobilisation allows exploitation of special properties that occur at this size, whilst the nanoporous support may provide a useful transport vehicle. Combining highly active nanoparticle centres with the nanopore environment, generates specific adsorption sites, partitioning the exterior and the interior pore structure, inhibiting nanoparticle aggregation and restricting nanoparticle growth to a particular size regime (potentially directed by support media pore size). MNP size control is normally achieved via the introduction of hydrophobic capping agents (e.g., HS–C*_n_*H_2_*_n_*_+1_) [6,7], but the use of a green polar solvent like H_2_O is problematic in this context. Although alternative solvents such as supercritical CO_2_ have been successfully employed [8–10], the use of CO_2_-philic surfactants increases costs and presents problems with regard to MNP recovery. The second concern in a green MNP preparation method is the choice of reducing agent and synthetic media, as conventional approaches typically employ organic solvents and toxic reducing agents (e.g., NaBH_4_ or N,N-dimethylformamide) [11], presenting environmental problems associated with safety and waste disposal.

Silver nanoparticles (AgNP) are a particularly interesting class of MNP, desirable in applications such as antimicrobial surfaces or wound dressings [12,13] or healing [14], where activity is determined by MNP size and density of states, allowing the existence of Ag^+^; the active cation in antimicrobial behaviour [15,16]. AgNP have attracted much attention in biomedical applications, and have been used as optical indicators for (bio)molecules [17,18], and cytoprotective agents against HIV-1 infected cells [19]. Liquid phase dispersions of AgNP, whilst providing small nanoparticle sizes, are not conventionally suitable for the aforementioned applications [20]. Therefore a solid support must be used to stabilise nanoparticles to the active size range (e.g., diameters <50 nm) and as a transfer vehicle to the desired application environment. The use of inorganic supports [21,22] or carbon nanotubes [23,24], are not only costly but arguably rely on further derivitisation to achieve the necessary biocompatibility and safety for the intended patient. Use of polysaccharides to stabilise MNP is an interesting inexpensive option, particularly if the hydroxyl rich surface can be exploited as support medium and reducing agent in tandem. Utilisation of non-porous versions of (soluble) starch [25,26], chitosan (derivatives) [27–29], and cellulose [30,31] in the synthesis of AgNP have recently been reported. However, this is arguably not an efficient use of the polysaccharide, as in the native non-porous state, hydroxyl group accessibility is limited, reducing the overall potential for AgNP loading, and resource utilisation, whilst a lack of pore structure provides no scope for size control and stabilisation. An accessible porous polysaccharide structure is also advantageous as it would present a hybrid system intermediary between more classical “soft” organic materials and “hard” inorganic systems.

In this context nanoporous polysaccharides (e.g., mesoporous starch (MS) [32] or pectin [33]) present a high surface area and (meso)porous framework within which nanoparticle growth may potentially be limited to a desired size regime, enabling improved mass transfer kinetics of the active species (e.g., Ag^+^) to the application environment. Furthermore, in many applications such as wound dressings or purifying agents, AgNP must be easy to handle and the applied material recoverable. A solid polysaccharide presents a useful nanoparticle support in this respect; biocompatible, insoluble under ambient or biological conditions, and easily recovered. The use of nanoporous polysaccharides in turn should allow direction of AgNP size and stabilisation and therefore enhanced activity. Herein, we present the sustainable preparation, characterisation and application of MS confined AgNP materials (MS/AgNP), demonstrating excellent antimicrobial activity against both Gram positive (*S. aureus*) and negative (*E. coli*) model bacteria.

## 2. Results and Discussion

### 2.1. Characterisation of MS/AgNP Materials

**Figure 1:**
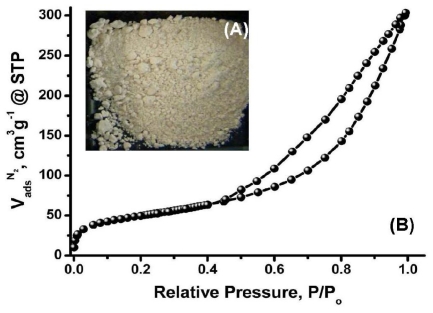
(**A**) Photograph; and (**B**) Representative N_2_ sorption profile of MS/AgNP material (Ag loading: 0.180 mmol g^−1^).

**Figure 2:**
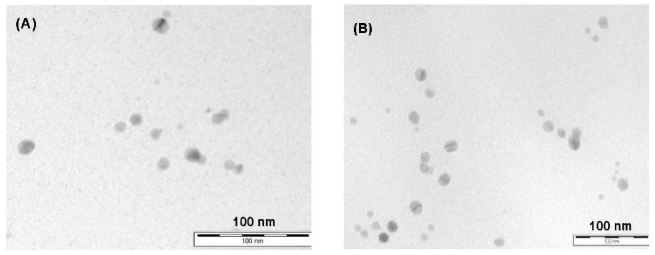
Representative TEM micrographs of MS/AgNP materials; Ag loading (**A**) 0.059 mmol g^−1^; and (**B**) 0.180 mmol g^−1^.

**Figure 3:**
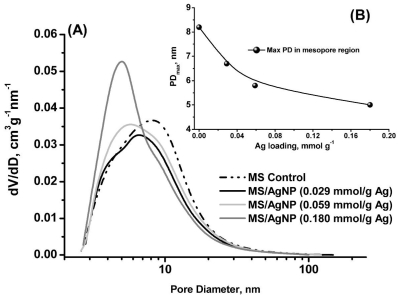
(**A**) Pore size distributions of MS and MS/AgNP materials prepared at increasing Ag loading; and (**B**) Relationship between pore diameter maximum in mesopore region and Ag loading.

MS/AgNP materials were a beige/yellow colour, indicative of the formation of AgNP species (Figure 1 (A)) [34]. TEM images were initially used to confirm the existence of AgNP within the MS support and aid description of the size, shape and dispersity of the supported MNP system. All MS/AgNP materials presented nanoparticle sizes in the range *ca.* 10–25 nm (independent of Ag loading) (Figure 2). AgNP size produced via the route reported here are comparable with literature examples in terms of size and dispersity within the biodegradable polysaccharide network [21]. To characterise and describe the surface and porous properties as influenced by AgNP loading, N_2_ sorption analysis was performed (Table 1). All samples presented high surface area (*S*_BET_ > 150 m^2^ g^−1^) and mesopore volumes (*V*_meso_> 0.47 cm^3^ g^−1^). Type IV (H3) N_2_ sorption profiles (Figure 1 B and Figure S1 ESI) were typical for all samples, indicative of a slit shaped pore structure, comparable to previously reported data [32,35,36]. Upon increasing Ag loading, textural properties as compared to the control MS sample showed no real trend in terms of a change in surface area or mesopore volume, and remained relatively constant. However, pore size distributions compared to the control MS sample, showed that with increasing Ag loading, rather than an anticipated consistent pore volume shrinking, the pore size distribution in fact becomes increasingly narrower and more classically Gaussian-like, suggesting some cooperative assembly between the growing AgNP and nanoporous polysaccharide network (Figure 3 (A)).

The pore diameter maxima in the mesopore region were observed to be equally sensitive to Ag loading, reducing in size in a near-linear manner, presumably as pores in the upper mesopore region become filled with larger AgNP (Figure 3 B). This behaviour is in contrast with previously reported MS/PdNP materials, which showed a gradual decrease in all textural properties as a consequence of PdNP formation at increasing loading [37]. Nanoparticle preparation was performed here conveniently in the aqueous phase due to the limited solubility of AgNO_3_ in other solvents used in the preparation of MS. It was anticipated that deposition of Ag^+^ would occur at hydroxyl rich polysaccharide pore wall sites, where reduction is believed to initiate. In this context Wallen *et al.* have speculated that Ag^+^ (aq) may play a role in guiding polysaccharide supramolecular organisation [25,26], whilst Vigneshwaran *et al.* have proposed that AgNP may be stabilised within the helical structure of the soluble starch amylose component [20].

Interestingly for the system reported here, AgNP growth within the mesopores was observed to enhance the uniformity of polysaccharide network upon increasing AgNP loading, as reflected in the narrowing pore size distributions. In this regard it is important to note that both the total pore volume and average pore diameters of the as-synthesised MS/AgNP materials are influenced by the increasing “Ag” content, but in fact it appears that it is the diameter maximum in the pore size distribution becoming increasingly smaller and the overall distribution increasing narrower and sharper (Figure 3). Under the synthetic conditions reported here, the hydrogen bonded porous polysaccharide gel network is presumably responsive to changes in the ionic potential of the system (e.g., increase in AgNO_3_), and therefore adapts itself to accommodate increasing MNP size, driving the system to an alternative metastable energy state, in turn promoting increased uniformity of the mesopore size.

We propose that in the initial stages of the addition of Ag^+^ to the polysaccharide gel, the silver cations are adsorbed within the recrystallised starch (amylose) blocks, due to specific interaction of silver with functional groups of starch, whilst the nitrate counter anions presumably interact with the “block” surface (Figure 2S ESI). Repulsive forces, for example between negative charged blocks, stabilise the interblock separation distance which becomes increasingly more uniform with increasing silver system content. The effect of the solvent exchange step is to essentially fix this structure (*i.e.*, to decrease starch (amylose) segmental mobility), and in turn the silver cations are reduced by the polysaccharide-nitric acid complex (e.g., nitric acid generates aldehydes as one co-product of starch oxidation). The atomic silver produced inside porous starch matrix potentially may migrate into the mesopore domains and the AgNP grows there as a result of aggregation.

Therefore the size of the nanoparticles is limited by diameter of pore in which they are forming, hence the AgNP sizes observed from TEM images are continuously uniform, and potentially become increasingly uniform with increasing Ag concentration as observed from a statistical particle size analysis (Figure 2). The observed stabilisation behaviour of the rather uniform mesoporous structure of the porous starch form by the specific adsorption of silver is potentially of great interest to the field of nanotechnology.

Under the synthetic conditions reported here, the hydrogen bonded porous polysaccharide gel network is presumably responsive to changes in the ionic potential of the system (e.g., increase in AgNO_3_), and therefore adapts itself to accommodate increasing MNP size, driving the system to an alternative metastable energy state, in turn promoting increased uniformity of the mesopore size. This inexpensive and simple polysaccharide gel based route to control the size uniformity of AgNP is promising and also potentially indicates that the formed AgNPs are robust to initial silver nitrate concentration. Moreover, this mechanism also suggests the potential for very high loadings of atomic silver (*i.e.*, small nanoparticles/cluster) are successfully stabilised within the porous starch matrix.

**Figure 4:**
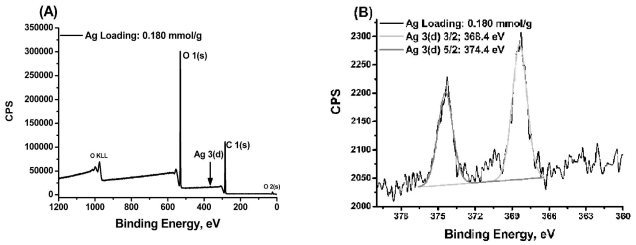
(**A**) XPS survey scan of MS/AgNP material; and (**B**) High resolution XPS spectra of the Ag 3 (d) photoelectron envelope (loading: 0.180 mmol g^−1^).

MS/AgNP materials were further characterised by XPS to analyse the Ag chemical state and confirm complete reduction (Figure 4). The XPS survey scans indicate the predominant chemical signatures of the polysaccharide support (*i.e.*, C 1(s) and O 1(s) photoelectron envelopes; Figure 4 (A)). Ag loading at the surface was not significant enough to enable quantification of Ag from the survey scan, indicating the development of AgNP buried deep inside the MS pore structure; (NB: *Ag loading was confirmed* via *elemental analysis*). High resolution XPS spectra of the Ag 3(d) photoelectron region for MS/AgNP materials, present peaks at 368.4 and 374.4 eV assigned to the Ag 3(d)_5/2_ and Ag 3(d)_3/2_, with relative intensities of 2:3, (typical for d electron orbitals) (Figure 4 (B)) [38]. As Ag loading increases, the relative intensity of the Ag 3(d) peaks increase accordingly, and peaks become more resolved from the baseline (Figure S3 SI), whilst the binding energies reported here are typical for those previously observed for the metallic Ag^0^ state [38,39]. Small variations in the binding energy may be attributed to Ag interacting with the electronegative oxygen-rich polysaccharide surface, resulting in a small distortion of the Ag e^−^ density [40]. Importantly XPS analysis did not indicate peaks associated with physisorbed AgNO_3_ (e.g., 367.0 and 373.0 eV), confirming the complete reduction to metallic Ag^0^ [38–40].

**Figure 5:**
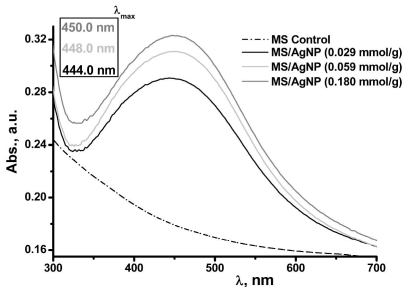
DRUVs analysis of MS/AgNP materials prepared at increasing Ag loading (*surface plasmon maxima* (*λ_max_*) *indicated*).

Further support for the presence of metallic AgNP comes from the characteristic surface plasmon resonance at *ca.* λ = 447.5 nm (±2.8 nm) in the DRUVS spectra for these MS/AgNP materials, with a broad absorption band in the range 320–600 nm (Figure 5), with a λ_max_ in the 444–450 nm region. As seen from TEM images (Figure 2), AgNP are relatively well-dispersed within the MS matrix, presenting spherical nanoparticles of diameters between 10–25 nm. Sanchez-Cortes *et al.* have previously reported that for 10 nm AgNP in solution, a well resolved surface plasmon resonance at λ_max_ ~400 nm is observed [41]. Here, the surface plasmon resonance band maxima for MS/AgNP was red-shifted and broader, compared to previously reported solution phase spectra by Sanchez-Cortes *et al.* and Kapoor [41,42]. Here the spectral red-shift occurs as a function of increasing Ag loading, although relatively small (*ca.* ~6 nm), changes in the electronic environment as a consequence of polysaccharide/AgNP interactions (and between adjacent AgNP), may result in alteration of the system dielectric properties.

### 2.2. Antimicrobial Activity of MS/AgNP Materials

**Figure 6:**
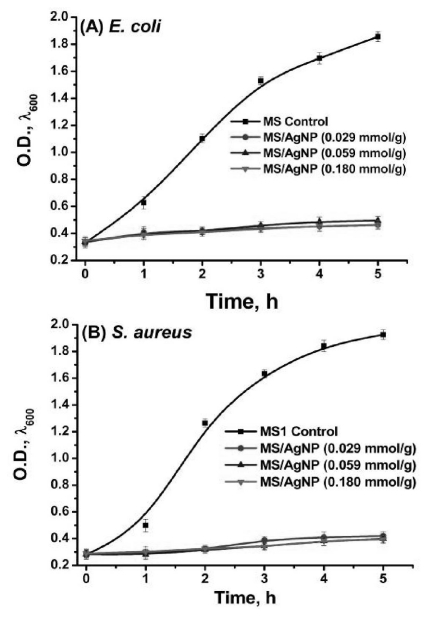
Bacterial growth curves in Lysogeny broth (LB) growth media, for (**A**) *E. coli*; and (**B**) *S. aureus* using MS/AgNP prepared at increasing Ag loading. Bacterial growth was monitored measuring the O.D.*_600_*.

AgNP are well known to exhibit antimicrobial properties, where Ag^+^ is thought to interact with sulfur or phosphorous containing cell matter (e.g., proteins or DNA), inhibiting cell function or replication [15,16]. The inhibitory effect of MS/AgNP materials were investigated during the aqueous phase growth cycle of *E. coli* (Gram negative) and *S. aureus* (Gram positive) model bacteria (Figure 6). Both *E. coli* and *S. Aureus* grew well in the presence of the MS control material. However, for both bacteria, MS/AgNP materials were shown to dramatically inhibit growth, even at very low Ag loadings (*i.e.*, 0.029 mmol g^−1^). Results were almost identical for all AgNP materials against *E. coli* (Figure 6 A), but at a loading of 0.029 mmol g^−1^ material appeared to be marginally less effective against *S. aureus*growth (Figure 6 B).

**Figure 7:**
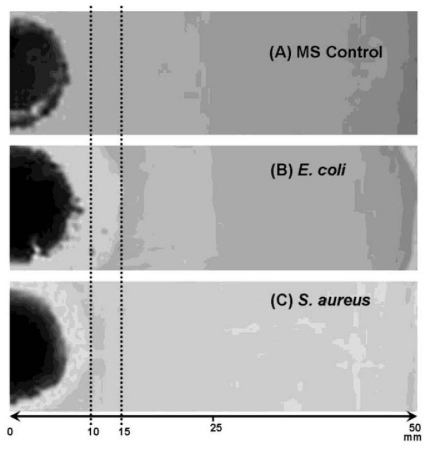
Representative photographs of agar growth plates for (**A**) *S. aureus* + MS control experiment; (**B**) *S. aureus* + MS/AgNP (0.180 mmol g^−1^); and (**C**) *E. coli* + MS/AgNP (0.180 mmol g^−1^) (Scale = 50 mm).

MS/AgNP materials were also tested for bacteria growth inhibition on agar growth plates to establish activity of the supported material in the dry state. In this case 50 mg of MS/AgNP material (e.g., Ag loading: 0.180 mmol g^−1^) was loaded onto the inoculated agar growth plate (Figure 7). Plates were then left to incubate overnight at 37 °C, to yield bacterial colonies on the plate surface grown in the presence of the MS/AgNP material (or the MS control sample (Figure 7 A). For samples incubated in the presence of the MS control sample, no inhibition was observed (Figure 7 A). MS/AgNP presented good growth inhibition independent of the Ag loading and appeared more active against *E. coli*, as demonstrated by increased inhibition ring distance (Figure 7 B and C).

This simple and green material preparation strategy renders materials active against these model bacteria. This is a promising result as in principle our approach can be extended to a range of our recently reported polysaccharide-derived mesoporous materials [4,32,33,36,43]. It is anticipated that AgNP size control and polydispersity may be enhanced through selection of different mesoporous polysaccharides or Starbon^®^ material [32,33,36,43]. Importantly, materials presented herein are stable under non-humid storage conditions and show no signs of AgNP aggregation even after 2 months storage, as evidenced by a consistent surface resonance plasmon peak from DRUVs spectra. Utilising this approach, plus the surface chemistry flexibility afforded by porous polysaccharide and Starbons^®^ material technology, the preparation of mesoporous monoliths or films will enable development in applications, such as bioactive films or surfaces (e.g., in food coatings) or direct integration in water filter/purification [33,36].

The MS support, whilst providing AgNP confinement and stabilisation, is transient and therefore in principle can be easily removed simply by heating in water (>100 °C) to solubilise and break down the hydrogen bond network, enabling the separation/controlled release of these AgNP. The accessibility of AgNP within the MS matrix, in principle will allow secondary use of these materials in heterogeneous catalysis [44,45], but also allows post-functionalisation strategies to be performed on the AgNP prior to any such removal—in this respect the MS support acts as an inexpensive, sustainable nanoparticle stabiliser. Therefore by selection and manipulation of the textural properties of the heterogeneous support, in unison with the reduction step, (e.g., via polysaccharide selection) it should be possible to exert still further control over size and shape of AgNP. This simple and sustainable AgNP synthesis strategy opens a wide range of opportunities, providing an inexpensive route to potentially revenue-rich applications.

## 3. Experimental Section

### 3.1. Materials and Chemicals

AgNO_3_ (≥99.0%) was purchased from Sigma-Aldrich (Gillingham, UK) and used as received. Ethanol (analytical grade) was purchased from Fisher Scientific (Loughborough, UK) used as received. Triple distilled water was used as prepared on site. High amylose corn starch (amylose content *ca.* ~75%) was used as the precursor starch.

### 3.2. Preparation of Mesoporous Starch Supported Ag Nanoparticle Materials

Mesoporous starch (MS) was prepared in the aqueous gel form as reported elsewhere [32,36,43]. A known quantity of MS gel (*ca.* 21.0 g, equating to 1.0 g of starch solid) was added to a 100 mL laboratory glass beaker with stirrer bar, and left to stir for 1 h. To the gel, 25 mL of AgNO_3_ (aq) of the desired concentration was added, and left to stir for 15 h at room temperature to allow for complete system homogenisation, yielding a beige/brown gel. The solvent exchange of water for ethanol (as reported elsewhere) [32,33] was then conducted until the system contained practically 100% ethanol. The material was then filtered and partially dried. The solid was then reimmersed in 100% ethanol and left to stir for 1 h. This washing step was repeated once more followed by filtration and drying under vacuum for 16 h at 60 °C.

### 3.3. Characterisation

N_2_ sorption analysis was performed on a Micromeritics ASAP 2010 porosimeter. Samples were degassed at 60 °C under vacuum (*p* < 10^−2^ Pa) for at least 3 h prior to analysis. Data processing was performed using ASAP 2010 version 5.02 and Origin 7.5 software. Sorption isotherms were measured at −196 °C. Specific surface areas (*S*_BET_) were determined via the BET method [46], using the cross sectional area of the nitrogen molecule (0.162 nm^2^), and a 10–20 point BET plot over the P/P_o_ range of 0.06–0.30, where a linear relationship was maintained. Average pore size (APD) and mesopore volume (*V*_meso_) were calculated using the BJH model [47]. Total pore volume (*V*_total_) was determined at a relative pressure of 0.975. X-ray photoelectron spectroscopy (XPS) spectra were recorded on a Kratos Axis Ultra DLD photoelectron spectrometer (Kratos Analytical, Shimadzu Corp., Japan) using a hemispherical photoelectron analyser, employing a monochromatic AlKα X-ray source (75–150 W) and analyser pass energies of 160 eV (for survey scans) or 40 eV (for high resolution scans). Samples were mounted using a double-sided adhesive tape and binding energies referenced to the C 1(s) binding energy of the adventitious carbon peak which was taken to be 285.0 eV. Prior to analysis samples were degassed overnight at ultrahigh vacuum (*P* < 5 × 10^−10^ Torr). XPS data analysis was performed on CasaXPS software (version 2.3.14) using default Schofield sensitivity factors, a linear background model and software default GL(30) (70% Gaussian, 30% Lorentzian) peak fitting mode. Diffuse reflectance ultra violet spectroscopy (DRUVs) was performed using a Jasco V550 UV/VIS spectrophotometer (Jasco, UK), equipped with a solid state diffuse reflectance mode analysis cell, and acquired in the 190–900 nm range, at a scanning speed of 100 nm min^−1^ and 0.5 nm data pitch. A Jasco supplied background polystyrene block was used as the spectral reference material. Transmission Electron Microscopy (TEM) was performed using a Tecnai 12 BioTwin transmission electron microscope (FEI Company, USA). Samples were suspended in acetone or ethanol, and then deposited onto carbon grids via solvent evaporation.

### 3.4. Investigation of Antimicrobial Properties of Porous Starch Supported AgNP Materials

#### 3.4.1. Procedure for Liquid Phase Growth Inhibition Studies

Bacteria used in this study were *Escherichia (E.) coli* strain W3110 and *Staphylococcus (S.) aureus* strain 8325–4. 1 mL of Lysogeny broth (LB) was inoculated with a sample of bacterial colony removed from a standard bacterial growth plate using a sterile wire inoculation loop. 10 μL of the resulting solution was injected into 10 mL of fresh LB media in a sterile sealable vessel. 25 mg of MS/AgNP material was then added, and an optical density measurement was taken for *t* = 0 h. Samples were then placed in a thermostated incubation room at 37 °C, and shaken at 200 r.p.m. to ensure continuous aeration of cultures. Optical density measurements at 600 nm (O.D._600_) were recorded at hourly intervals for 5 h. Liquid phase studies were performed in replicate.

#### 3.4.2. Procedure for Zone of Inhibition Plate Studies

1 mL of LB was inoculated with a sample of bacterial colony removed from a standard bacterial growth plate using a sterile wire inoculation loop. 50 μL of the resulting solution was placed onto a freshly prepared LB agar plate. The suspension was spread across the surface of the agar with a sterile glass spreader. A 1.0 cm diameter circular piece of LB agar was removed from the plate centre into which 50 mg of MS/AgNP was placed. The plates were then incubated at 37 °C for 24 h.

## 4. Conclusions

The preparation route to mesoporous starch confined Ag nanoparticles presented here is simple, green and efficient. Materials are prepared in a facile sustainable manner, based around the generation of porous carbohydrate-based polymeric structures, rendering materials antimicrobially active against model Gram positive and negative bacteria, (*E. coli* and *S. aureus*), even at low Ag loadings (<0.18 mmol g^−1^). The materials prepared are highly mesoporous and have high surface areas, with mesoporous domains of the MS support allowing a degree of size and dispersity control, limiting AgNP size to within a range of 10–25 nm.

The polysaccharide support is biocompatible and can be used directly in this form (under the test conditions used), as demonstrated by zone of inhibition testing. AgNP are stable and their size is comparable to size and dispersity of those produced using typical methods. The use of environmentally friendly and renewable materials as the reducing stabiliser and transport media (e.g., mesoporous starch), as well as the benign solvents used, offers numerous benefits ranging from environmental safety to the ready integration of these nanomaterials into biologically relevant systems. Use of a range of porous polysaccharides in this simple synthetic approach potentially will allow extension of the described method and further direction of AgNP size, and therefore activity [33,48]. This use of renewable polysaccharides/solvents, plus the continuing development of porous polysaccharide-derived materials, presents a number of opportunities for further development of green Ag nanoparticle synthesis, particularly with regard to applications in antimicrobial textile fibres or film surfaces.

## Supplementary Information



## Figures and Tables

**Table 1:** N_2_ sorption data for MS/AgNP materials prepared at increasing Ag loadings.

Ag Loading/mmol g^−1^	a*S*_BET_/m^2^ g^−1^	b*V*_total_/cm^3^ g^−1^	b*V*_meso_/cm^3^ g^−1^	c*V*_micro_/cm^3^ g^−1^	bAPD/nm	bPD_max_/nm
*0.000	170	0.53	0.50	0.008	9.6	8.2
0.029	152	0.47	0.44	0.007	8.8	6.7
0.059	161	0.49	0.45	0.007	9.0	5.8
0.180	175	0.47	0.45	0.008	7.7	5.0

[a] BET surface area using N_2_ adsorption data in the relative pressure range 0.05–0.2;

[b] BJH average pore diameter (PD), pore diameter maximum in mesopore region (PD_max_), mesopore (*V*_meso_) and total pore volume (*V*_total_);

[c] Micropore volume from the t-plot method.

* [MS = control experiment performed in the absence of AgNO_3_].
