# Supplementary material for: A Sweet Killer: Mesoporous Polysaccharide Confined Silver Nanoparticles for Antibacterial Applications
Source: Int J Mol Sci. 2011 Sep 9;12(9):5782–96. doi: 10.3390/ijms12095782 (PMC3189750; doi:10.3390/ijms12095782)
Supplement: Supplementary file 1 [file ijms-12-05782-s001.pdf]

Supplementary Information

Robin J. White [1,†,*], Vitaly L. Budarin [1], James W.B. Moir [2] and James H. Clark [1]

[1] Green Chemistry Centre of Excellence, Department of Chemistry, University of York, Heslington, York, YO10 5DD, UK; E-Mails: vlb1@york.ac.uk (V.L.B.); jhc1@york.ac.uk (J.H.C.)

[2] Department of Biology (Area 10), University of York, PO Box 373, York, YO10 5YW, UK; E-Mail: james.moir@york.ac.uk

[†] Current Address: Max-Planck-Institut für Kolloid-und Grenzflaechenforschung, MPI Campus Golm, Am Muehlenberg, 14476 Golm (Post: 14424 Potsdam), Germany.

* Author to whom correspondence should be addressed; E-Mail: robin.white@mpikg.mpg.de; Tel.: +49-0-331-567-9508.

Received: 12 August 2011 / Accepted: 23 August 2011 / Published: 9 September 2011

Abstract: Silver nanoparticles (AgNP) confined within porous starch have been prepared in a simple, green and efficient manner, utilising the nanoporous structure of predominantly mesoporous starch (MS) to act as nanoparticle stabiliser, support and reducing surface. MS/AgNP materials present high surface areas (S_{BET} > 150 m^2 g^{-1}) and mesopore volumes (V_{meso} > 0.45 cm^3 g^{-1}). The interaction of the AgNP precursor and forming nanoparticle nuclei with the mesoporous domains of the porous polysaccharide, direct porosity to increasingly narrower and more defined pore size distributions, indicative of a degree of cooperative assembly. Transmission electron microscopy images indicated the presence of spherical AgNP of a size reflective of the porous polysaccharide mesopore diameter (e.g., 5–25 nm), whilst XPS analysis confirmed the metallic Ag^0 state. Materials were prepared at relatively low Ag loadings (<0.18 mmol g^{-1}), demonstrating excellent antimicrobial activity in solid and liquid phase testing against Gram negative (*E. coli*) and positive (*S. aureus*) model bacteria. The resulting materials are biocompatible and present a useful solid porous carbohydrate-based polymer vehicle to control the AgNP size regime and facilitate transference to a biological environment.

Keywords: antibacterial; mesoporous; nanoparticles; nanotechnology; polysaccharide; silver; starch

---

Figure S1. N_2 sorption isotherm profiles for MS confined AgNP prepared at increasing loading; (A) MS control sample, (B) Ag loading 0.029 mmol g^{-1}, (C) Ag loading 0.059 mmol g^{-1}, and (D) Ag loading 0.180 mmol g^{-1}.

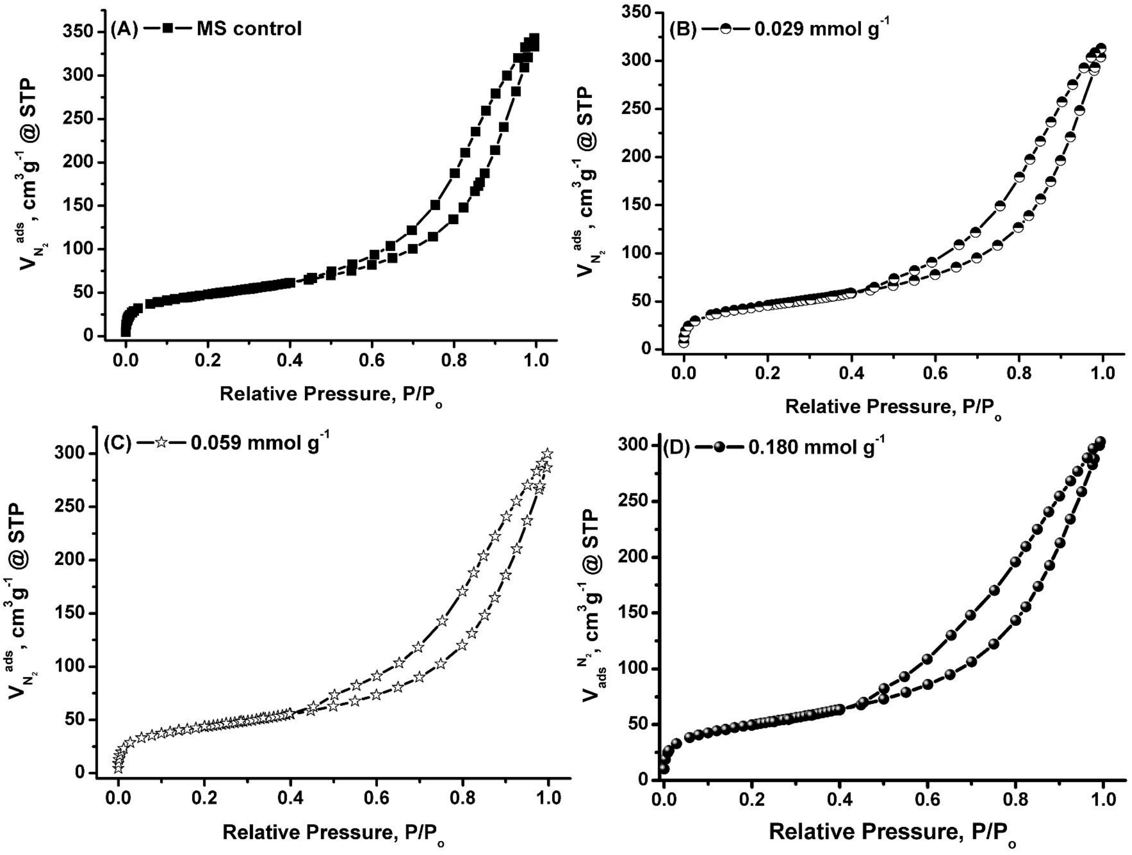

Figure S2. Representative schematic depicting the association and "self" organization of starch "amylose" blocks and $AgNO_3$.

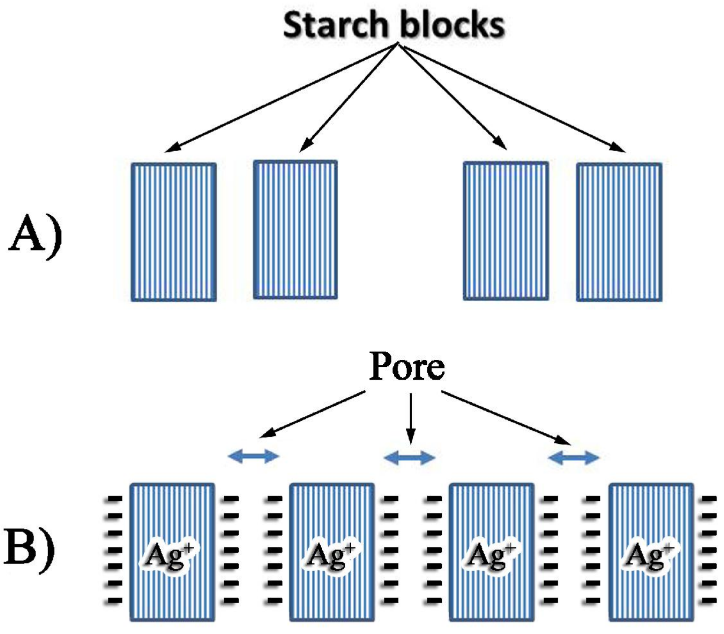

Figure S3. High resolution Ag 3(d) XPS spectra including spectral fittings for MS/AgNP materials prepared at increasing Ag loadings; (A) 0.029 mmol g^{-1}; (B) 0.059 mmol g^{-1} and (C) 0.180 mmol g^{-1}.

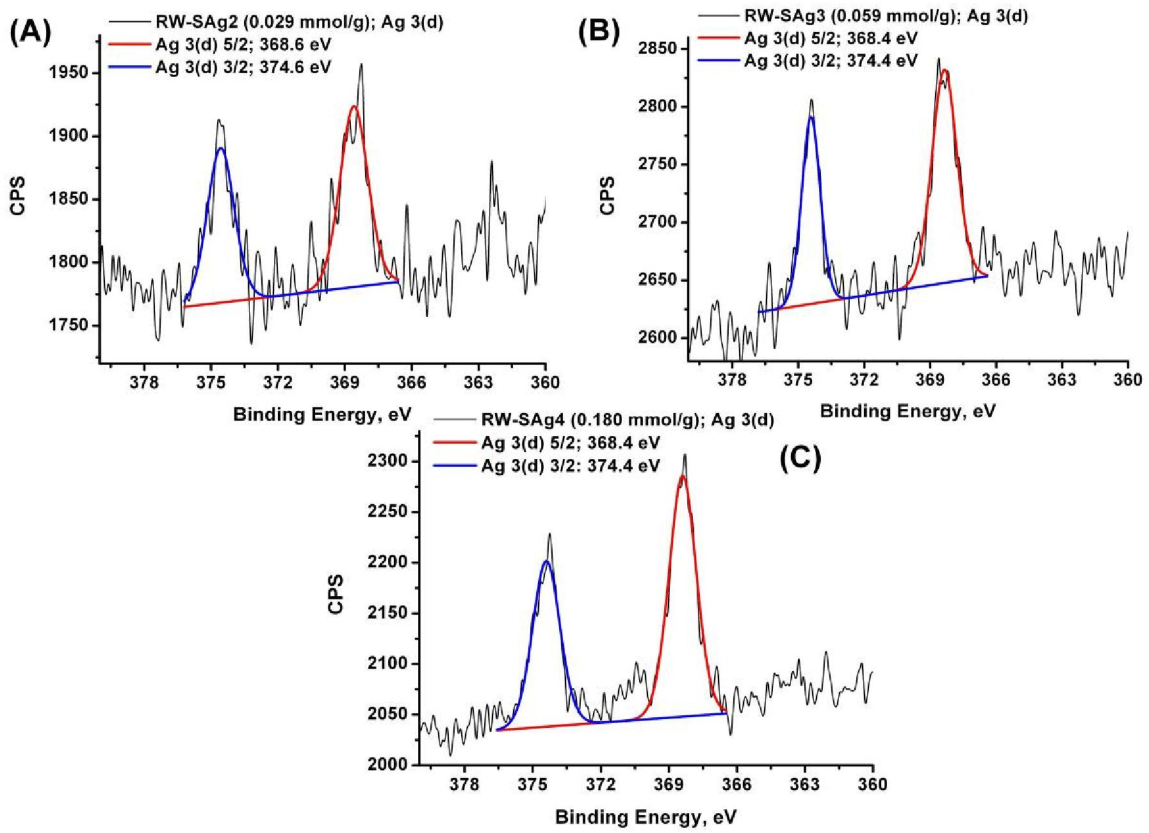

© 2011 by the authors; licensee MDPI, Basel, Switzerland. This article is an open access article distributed under the terms and conditions of the Creative Commons Attribution license (http://creativecommons.org/licenses/by/3.0/).
